# Associations of the activity and concentration of carbonic anhydrase VI with susceptibility to dental caries: A systematic review and meta‐analysis

**DOI:** 10.1002/cre2.723

**Published:** 2023-02-23

**Authors:** Rania Al‐Mahdi, Hesham Al‐Sharani, Mohammed Al‐Haroni, Esam Halboub

**Affiliations:** ^1^ Department of Clinical Dentistry, Faculty of Health Sciences UiT The Arctic University of Norway Tromsø Norway; ^2^ Centre for Public Health Data and Policy, National Center for Epidemiology and Population Health, ANU College of Health and Medicine The Australian National University Canberra Australia; ^3^ Department of Oral and Maxillofacial Surgery, College of Dentistry Ibb University Ibb Yemen; ^4^ Centre for New Antibacterial Strategies UiT The Arctic University of Norway Tromsø Norway; ^5^ Department of Maxillofacial Surgery and Diagnostic Sciences, College of Dentistry Jazan University Jizan Saudi Arabia; ^6^ Department of Oral Medicine, Oral Pathology and Oral Radiology, Faculty of Dentistry Sana'a University Sana'a Yemen

**Keywords:** CA VI activity, CA VI concentration, carbonic anhydrase VI, dental caries

## Abstract

**Objectives:**

A number of studies have claimed that carbonic anhydrase VI (CA VI) is associated with dental caries. The aim of this systematic review and meta‐analysis was to systematically review and analyze the literature on the association of CA VI (in terms of concentration and activity) with dental caries.

**Materials and Methods:**

A systematic review was conducted according to the Preferred Reporting Items for Systematic Reviews and Meta‐Analyses (PRISMA) guidelines. Relevant search terms were employed to search the following databases: PubMed, Web of Science, Scopus, China National Knowledge Infrastructure (CNKI), and Cochrane Library databases. Eligible publications from inception to August 2022 were included. The relevant records were assessed independently by two reviewers, and a meta‐analysis was performed using RevMan 5.3.

**Results:**

Out of 237 relevant records from the initial search, 9 met the criteria for this review. The 9 papers, including 477 participants, were qualitatively analyzed. Seven studies with 411 participants (203 caries‐free) were included in the meta‐analysis on CA VI activity, and 2 studies with 141 participants (71 caries‐free) were included in the meta‐analysis on CA VI concentration. The results showed that CA VI activity was significantly higher among participants with caries than their caries‐free counterparts (standardized mean difference (SMD) = 0.894, 95% confidence interval (CI_95%_): 0.386 and 1.392; *p* < 0.001), whereas the CA VI concentration was significantly lower among participants with caries than their caries‐free counterparts (SMD = −0.672, CI_95%_: −1.011 and −0.332; *p* < 0.001).

**Conclusions:**

This meta‐analysis of a relatively small number of studies suggests that the CA VI concentration is lower and CA VI activity is higher in patients with dental caries than in caries‐free individuals; however, further studies are needed to determine the exact role of CA VI in dental caries.

## 1 INTRODUCTION

Dental caries is one of the most prevalent chronic diseases worldwide; individuals are susceptible to this disease throughout their lifetime. The aetiology of dental caries includes physical, biological, environmental, behavioral, and lifestyle‐related factors such as high numbers of cariogenic bacteria, inadequate salivary flow, insufficient fluoride exposure, and poor oral hygiene (World Health Organization, 2010). The salivary flow rate and salivary buffer capacity are considered important host factors that modify the dynamics of dental caries processes and are considered indicators of high dental caries risk (Leone & Oppenheim, 2001) if the amount and composition of the saliva results in the dissolution of dental hard tissues under the acidic conditions prevailing beneath dental plaque (Dodds et al., 2015). Previous investigations have suggested that some salivary proteins may be used as biomarkers for higher risk of dental caries (Roa et al., 2008; Tulunoglu et al., 2006). Carbonic anhydrase VI (CA VI) is one such protein that influences dental caries dynamics (Esberg et al., 2019; Kimoto et al., 2006).

CA VI is the main salivary zinc metalloprotein responsible for salivary pH homeostasis and regulation of buffer capacity by catalyzing the hydration of carbon hydroxide in the following reaction: CO_2_ + H_2_O ↔ HCO_3_^−^+ H^+^. CA VI is the only secreted isoenzyme of the carbonic anhydrase family and is mainly secreted by serous acinar cells in the parotid and submandibular glands (Fernley et al., 1995; Kivelä et al., 1999). CA VI is suggested to participate in several essential activities affecting oral health, such as the caries process, periodontal problems, and the sensation of a bitter taste (Arabacı et al., 2015; Esberg et al., 2019; Kimoto et al., 2006; Patrikainen et al., 2014). In addition, CA VI is suggested to contribute to the neutralization of biofilm acids because such buffering is mainly performed by bicarbonate; this neutralization could assist in the prevention of dental caries (Lima‐Holanda et al., 2021). Of the salivary proteins, CA VI performs an important role in several physiological processes, particularly oral homeostasis and dental tissue protection, and has been described as relevant to dental caries dynamics (Piekoszewska‐Ziętek et al., 2017).

Previous studies have analyzed the relationship between CA VI and dental caries, especially early dental caries, in children. These studies have focused on the concentration or activity of CA VI (or both), either in saliva or in biofilms (Borghi et al., 2017; Frasseto et al., 2012; Kivelä et al., 1999; Kormi et al., 2020; Öztürk et al., 2008; Picco et al., 2017, 2019; de‐Sousa et al., 2021a; de‐Sousa et al., 2021b). Many studies have reported inconsistent results regarding the association of the CA VI concentration with dental caries (Öztürk et al., 2008; Picco et al., 2017, 2019). However, more similar results have been reported in regard to CA VI activity (Borghi et al., 2017; Picco et al., 2017, 2019; de‐Sousa et al., 2021a; de‐Sousa et al., 2021b). Secretion of CA VI into the saliva is known to exhibit a circadian cycle, with its concentrations being very low during sleep and rising rapidly to the daytime level upon awakening and breakfast consumption (Parkkila et al., 1995). Accordingly, in most studies, saliva samples were collected early in the morning. Moreover, no report was found regarding the possible relationship between the activity and concentration of CA VI.

To our knowledge, no previous meta‐analysis has assessed the effects of the concentration or activity of CA VI on dental caries. Recently, a review suggested a potential association between CA VI and dental caries. However, this review had several drawbacks. First, they included only five studies, which were relatively old. Second, these studies included only children between 2 and 12 years old. Third, the effects of CA VI concentration and activity were not disentangled (Picco et al., 2022). Accordingly, this meta‐analysis aimed to synthesize the evidence of the effects of CA VI concentrations or activity on dental caries.

## 2 MATERIALS AND METHODS

### 2.1 Research question

The Preferred Reporting Items for Systematic Reviews and Meta‐Analysis (PRISMA) guidelines were rigorously followed in the present meta‐analysis (Moher 2009). The project was registered in the PROSPERO database (ID = CRD42021286508). The study question was as follows: “Is there any link between the concentration and/or activity of CA VI and the susceptibility to dental caries?”

### 2.2 Search strategy

The following databases were searched from inception to August 2022: PubMed, Scopus, Web of Science, China National Knowledge Infrastructure (CNKI), and Cochrane Library databases. The following keywords (or their Chinese translations) were searched: (“dental decay” OR “teeth decay” OR “teeth caries” OR “tooth caries” OR “tooth carious, lesion” OR “decay, dental” OR “carious lesions” OR “carious lesion” OR “lesion, carious” OR “lesions, carious” OR “caries, dental” OR “carious dentin” OR “carious dentins” OR “dentin, carious” OR “dentins, carious”) AND (“gustin” OR “carbonic anhydrase 6” OR “CA6 protein, rat” OR “carbonic anhydrase 6, rat” OR “gustin protein, rat” OR “CA6 protein, human” OR “carbonic anhydrase 6, human” OR “CA6 protein, human” OR “carbonic anhydrase VI, human”).

### 2.3 Eligibility criteria

All cross‐sectional and observational studies that investigated the possible associations of the concentration and/or activity of CA VI with the susceptibility of dental caries and met the following criteria were included in this systematic review: (1) included patients with dental caries and healthy controls (caries‐free), (2) had quantitative primary outcome measures (activity and/or concentration of salivary or plaque CA VI reported as the mean and standard deviation), and (3) published in English or Chinese. The exclusion criteria were as follows: (1) studies that lacked a control group, (2) studies that lacked necessary quantitative data (mean and SD), (3) studies published in other languages, (4) case series, (5) case reports, (6) in vitro studies, or (7) review articles.

### 2.4 Data extraction

Two authors (R. A. M. and H. A. S) independently extracted the following relevant information from the included studies: authors and year of publication, country, research design, number of cases and controls, participant sex and age, diagnostic criteria/tools for identifying dental caries, calibration of caries detection/diagnosis, evaluation techniques, assay method and unit of measurement for CA VI and the main outcomes (activity and concentration of CA VI).

### 2.5 Quality assessment

The Newcastle–Ottawa Scale (NOS) (Stang, 2010) was used to assess the risk of bias in the included studies. The NOS score is based on three primary components: study group selection (0–4 stars); comparability of cases and controls, by adjusting for relevant characteristics (0–2 stars); and exposure (0–3 stars). The overall quality of a given study was categorized as high (7 stars or more), moderate (4–6 stars), or low (0–3 stars). The above assessment was performed independently by two investigators (R. A. M. and H. A. S.). Any disagreement between the two investigators was resolved through discussion and/or consultation with a third reviewer (M. A. H.).

### 2.6 Statistical analyses

Comprehensive Meta‐Analysis software version 2.2.046 (Biostat) was used for the meta‐analysis. The standardized mean difference (SMD) along with the 95% confidence interval (CI) was calculated. To identify potential variability among studies, heterogeneity was identified according to the *χ* ^2^ test and *I* ^2^ statistics. The random‐effects model was used in cases of significant heterogeneity (*I* ^2^ > 50%), and the fixed‐effects model was used in cases of nonsignificant heterogeneity (*I* ^2^ ≤ 50%). A *p* value less than 0.05 was considered statistically significant.

### 2.7 Publication bias

Funnel plots and Egger's test were used to investigate publication bias. Review Manager 5.3 software (RevMan 5.3; The Cochrane Collaboration) was used to generate the funnel plots, while Stata for Windows was used to conduct Egger's test (version 15.1; Stata Corporation).

## 3 RESULTS

### 3.1 Study selection

**Figure 1:**
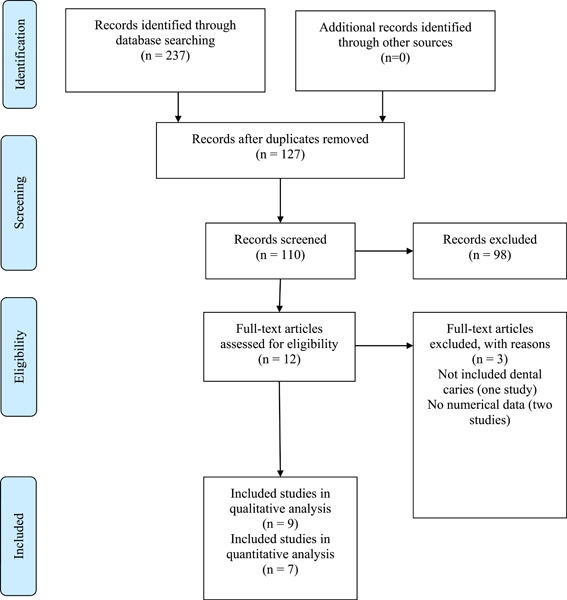
Schematic Preferred Reporting Items for Systematic Reviews and Meta‐Analyses flowchart of the literature search.

**Figure 2:**
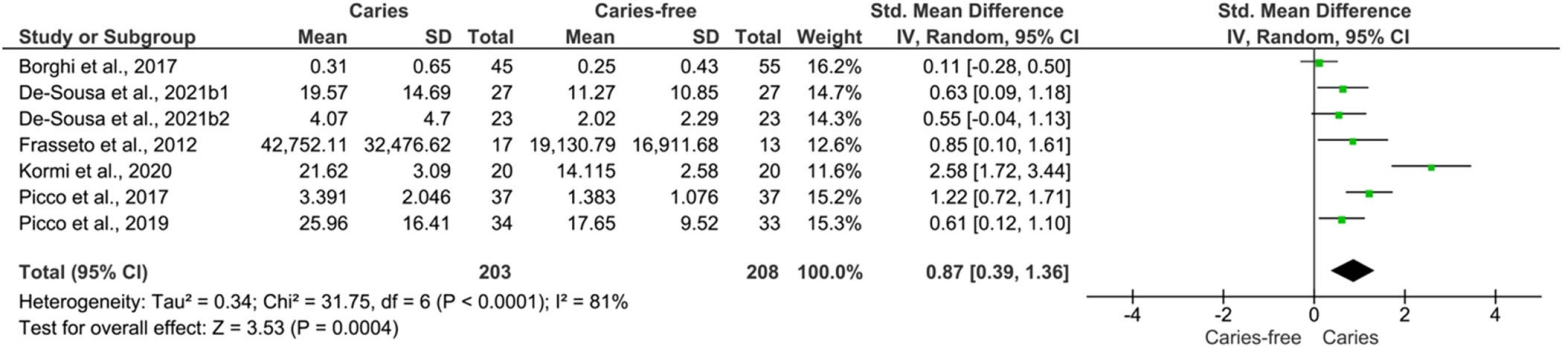
Forest plot assessing the difference in CA VI activity between caries and caries‐free participants. CA VI, carbonic anhydrase VI; CI, confidence interval; P, *p* value; SD, standard deviation.

As illustrated in Figure 1, the search of the five databases resulted in 237 potentially eligible studies. Among these, 127 were duplicates and were removed. The titles and abstracts of the remaining 110 studies were screened; 98 studies were found to be irrelevant and were removed. The full texts of the remaining 12 studies were comprehensively examined, and three studies were excluded; one study did not address dental caries, and the other two studies did not report numerical data (Leinonen et al., 1999; Szabó 1974; Wen & Que Guoying, 2018) (Supporting Information: Table 1). Thus, nine studies fulfilled the inclusion criteria for this systematic review and were included in the qualitative analysis (Borghi et al., 2017; Cardoso et al., 2017; Frasseto et al., 2012; Kivelä et al., 1999; Kormi et al., 2020; Picco et al., 2017, 2019; de‐Sousa et al., 2021a; de‐Sousa et al., 2021b). However, only seven studies were eligible for the quantitative analysis (meta‐analysis) (Borghi et al., 2017; Frasseto et al., 2012; Kormi et al., 2020; Picco et al., 2017, 2019; de‐Sousa et al., 2021b). Notably, the study by de Sousa et al. (2021b) in Figure 2 was considered to represent two separate studies. de‐Sousa et al., 2021b1; de‐Sousa et al., 2021b2, as it presented data from two different samples.

### 3.2 General characteristics of the included studies

Table 1 presents the detailed characteristics of the included studies. All the included studies were conducted in Brazil (Borghi et al., 2017; Cardoso et al., 2017; Frasseto et al., 2012; Picco et al., 2017, 2019; de‐Sousa et al., 2021a; de‐Sousa et al., 2021b), except Kormi et al. (2020), which was conducted in Saudi Arabia, and Kivelä et al. (1999), which was conducted in Finland. The included participants were 477 healthy children with an age range of 2 to 9 years, except the participants in Kivelä et al. (*n* = 209), who ranged in age from 18 to 24 years (Kivelä et al., 1999). The participants were divided into two groups based on whether they had dental caries (the decayed, missing, and filled teeth, related to deciduous/permanent tooth caries [dmft/DMFT] > 0; *n* = 234) or were caries‐free (dmft/DMFT = 0; *n* = 243). Caries status was identified using the dmft/DMFT score (either for teeth or for surfaces) in eight of the included studies; Cardoso et al. did not report using the dmft/DMFT score (Cardoso et al., 2017). Caries were diagnosed by one examiner in eight studies; Kormi et al. did not report how caries were diagnosed (Kormi et al., 2020). Only De‐Sousa et al. reported the calibration of the examiner regarding caries diagnosis (de‐Sousa et al., 2021b; de‐Sousa et al., 2021a). Eight studies (Borghi et al., 2017; Cardoso et al., 2017; Frasseto et al., 2012; Kivelä et al., 1999; Kormi et al., 2020; Picco et al., 2017; de‐Sousa et al., 2021a; de‐Sousa et al., 2021b) assessed CA VI activity either with zymography or other assays. The concentration of CA VI was assessed in only two studies (Picco et al., 2017, 2019). The activity and concentration of CA VI were assessed in stimulated salivary samples (Cardoso et al., 2017; Frasseto et al., 2012; Picco et al., 2017; de‐Sousa et al., 2021a; de‐Sousa et al., 2021b), unstimulated saliva (Borghi et al., 2017; Kormi et al., 2020), or dental biofilms (Picco et al., 2019; de‐Sousa et al., 2021a; de‐Sousa et al., 2021b).

**Table 1:** General characteristics of the included studies.

References	country	Study design	Participants				Diagnostic criteria/tool of dental caries	Calibration of caries diagnosis	Assay method and unit of measurement of CA VI	Saliva sample (S/US), and/or other source	Other outcomes measured
			All number age (mean) M/F	Cases number age (mean) M/F	Controls number age (mean) M/F	Comments					
Frasseto et al. (2012)	Brazil	Cross sectional (NR)	30 (45.3–80.3) months	17 (62.88 ± 17.5) months M/F = 7/10	13 (57.2 ± 87.2) months M/F = 8/5	Healthy	dmfs	One examiner (intraexaminer)	Activity: Zymography (Pixels area)	Stimulated saliva	Dental biofilm pH, salivary flow rate
Cardoso et al. (2017)	Brazil	Follow‐up	22 11–22 (14.8 ± 2.9) years M/F = 10/12	9 (developed caries after 6 months of follow‐up)	13 (remained caries free after 6 months of follow‐up)	Healthy underwent orthodontic treatment	Nyvad's caries detection criteria	One examiner (intraexaminer)	Activity: Zymography (Pixels area)	Stimulated saliva	Salivary flow rate, pH, and buffering capacity
Kormi et al. (2020)	Saudi Arabia	Cross sectional (NR)	80 6–8 (6.77 ± 2.1) years M/F = 46/34	Healthy + caries = 20 Asthma + caries = 20	Healthy + no caries = 20 Asthma + no caries = 20	Healthy and asthmatic children	dft/DMFT	NR	Zymographic method using a Human CA VI ELISA kit	Unstimulated saliva	Salivary pH
Kivelä et al. (1999)	Finland (Not explicitly reported)	Cross sectional (NR)	209 18–24 years (19.8) All males	Low CA VI = 66 Medium CA VI = 83 High CA VI = 60			DMFT	One examiner (reliability not reported)	Specific time‐resolved immunofluorometric assay	Stimulated saliva	Saliva secretion rate, pH, buffering capacity, microbial test, and amylase activity
de‐Sousa et al. (2021a)	Brazil	Cross sectional	44 4–5 years M/F ‐	22 (4.59 ± 0.50) years M/F = 18/4	22 (4.55 ± 0.60) years M/F = 7/15	Healthy	dmfs + the diagnosis of active white spot lesion	One examiner (intra and inter examiner)	Activity: Zymography (Pixels area)	Stimulated saliva and dental biofilm	Salivary pH, and buffering capacity. Dental biofilm pH, and buffering capacity
Picco et al. (2019)	Brazil	Cross sectional	74 7–9 years M/F ‐	34 7–9 years M/F ‐	33 7–9 years M/F ‐	Healthy	DMFT	One examiner (intraexaminer)	Activity: (pixels area) Concentration: Elisa (ng/µL)	Dental biofilm	Dental biofilm pH
Borghi et al. (2017)	Brazil	Longitudinal study (cross sectional in relation to CA VI data)	100 24–48 months female (44.6%) and of ethnicity not White (54.5%)	45 24–48 months M/F ‐	55 24–48 months M/F ‐	Healthy	dmfs dmft	One examiner (intraexaminer)	Activity: Zymography (pixels area)	Unstimulated saliva	alpha amylase activity and dental biofilm
Picco et al. (2017)	Brazil	Cross sectional	74 7–9 years M/F ‐	37 7–9 years M/F ‐	37 7–9 years M/F ‐	Healthy	DMFT	One examiner (intraexaminer)	Activity: Zymography (pixels area) Concentration: Elisa (ng/µL)	Stimulated saliva	Salivary pH, buffering capacity and flow rate
de‐Sousa et al. (2021b)	Brazil	Observational and cross‐sectional research	Study (1) 54 Study (2) 46 4–5 years M/F ‐	Study (1) 27 4.61 (0.63) years M/F = 1.54/1.0 Study (2) 23 4.55 (0.60) years M/F = 0.69/1:0	Study (1) 27 4.61(0.57) years M/F = 1.33/1.0 Study (2) 23 4.59 (0.50) years M/F = 1.75/1.0	Healthy	dmfs	One examiner (Intra‐ and inter examiner)	Activity: Zymography (Pixels/mg)	Study (1) Stimulated saliva Study (2) Dental biofilm	Salivary pH, buffering capacity and flow rate & dental biofilm pH, and buffering capacity

Abbreviations: CA VI, carbonic anhydrase VI; dft, decay and filled for deciduous teeth; dmfs, the decayed, missing and filled surfaces for deciduous teeth; dmft/DMFT, the decayed, missing, and filled teeth, related to deciduous/permanent tooth caries, respectively; F, females; M, males; NR, not reported numerically; SD, standard deviation.

### 3.3 Meta‐analysis results

**Figure 3:**
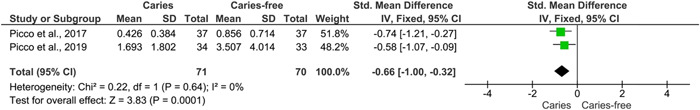
Forest plot assessing the difference in CA VI concentration between caries and caries‐free participants. CA VI, carbonic anhydrase VI; CI, confidence interval; P, *p* value; SD, standard deviation.

The numerical data on the main outcomes are presented in Table 2. Seven studies (Borghi et al., 2017; Frasseto et al., 2012; Kormi et al., 2020; Picco et al., 2017, 2019; de‐Sousa et al., 2021b) that assessed and reported numerical data on the activity of CA VI were included in the meta‐analysis (Figure 2). Furthermore, a meta‐analysis was performed including two studies that assessed and reported numerical data on the concentration of CA VI (Figure 3). All seven studies (Borghi et al., 2017; Frasseto et al., 2012; Kormi et al., 2020; Picco et al., 2017, 2019; de Sousa et al., 2021b) reported higher activity of CA VI among participants with caries than among their caries‐free counterparts (Figure 2). In contrast, the two studies (Picco et al., 2017, 2019) that reported the concentration of CA VI found lower levels among participants with caries than their caries‐free counterparts (Figure 3).

**Table 2:** Summary table of the main outcomes.

Study	Caries (cases)	CA VI activity/concentration cases	CA VI activity/concentration Control		Comments
Frasseto et al. (2012)	NR	Activity = 42,752.11 ± 32,476.62	Activity = 19,130.79 ± 16,911.68		Variation of salivary CAVI activity and child's age are associated with dental caries in preschool children.
Cardoso et al. (2017)	NR	NR numerically	NR numerically		Saliva of individuals under orthodontic treatment is subjected to changes in properties that have implications on the onset of active caries lesions.
Kormi et al. (2020)	NR	Levels healthy = 21.62 ± 3.09 Asthma = 7.03 ± 1.17	Levels healthy = 14.12 ± 2.58 Asthma = 12.27 ± 1.18		CA VI may serve as a protective mechanism against dental caries in children with bronchial asthma.
Kivelä et al. (1999)	7.3 ± 0.4 for whole sample	5 ± 2.89 mg/L for the whole sample, the level decreased with decreased DMFT			Low salivary CA VI concentrations are associated with increased caries prevalence, particularly in subjects with neglected oral hygiene.
de‐Sousa et al. (2021a)	dmft = 5.27 ± 5.90 WSL = 6.95 ± 4.82 Both = 12.23 ± 7.98	NR for both saliva and dental biofilm		NR for both saliva and dental biofilm	CA VI activity was significantly higher in saliva and biofilm of children with early childhood caries compared with caries‐free children
Picco et al. (2019)	DMFT 3.162 ± 1.385	Activity = 25.96 ± 16.41 Concentration = 1.693 ± 1.802		Activity = 17.65 ± 9.52 Concentration = 3.507 ± 4.014	CA VI activity was significantly higher in biofilm of children with caries than in the caries‐free children, while CA VI concentration was significantly higher in the biofilm of caries‐free children than in the caries group.
Borghi et al. (2017)	dmfs 10.24 ± 14.04 dmft 5.72 ± 4.57	Activity = 0.31 ± 0.65		Activity = 0.25 ± 0.43	Dropouts: after 1 year, 19 children were excluded due to preschool changes. The activity of the CA VI was significantly higher in saliva of children with caries than in those caries‐free (*p* ≤ .05).
Picco et al. (2017)	DMFT 3.162	Activity = 3.391 ± 2.046 Concentration = 0.4255 ± 0.3835		Activity = 1.383 ± 1.076 Concentration = 0.8561 ± 0.7141	A moderate positive correlation between CA VI activity and concentration was noted in the caries group. The salivary CA VI concentration was significantly higher in caries‐free children and the salivary CA VI activity was significantly higher in caries‐active children.
de‐Sousa et al. (2021b)	Study (1) dmfs ECC median = 8.00 (13.0) CF median = 0 Study (2) dmfs ECC median = 11.00 (12.0) CF median = 0	Study (1) Activity average 19.57 (14.69) Study (2) Activity average 4.07 (4.70)		Study (1) Activity average 11.27 (10.85) Study (2) Activity average 2.02 (2.29)	CA VI activity was higher in children with early childhood caries compared with caries‐free children. Saliva: No difference observed between groups when it comes to carbohydrates. Biofilm: No difference observed between groups when it comes to carbohydrates.

Abbreviations: CA VI, carbonic anhydrase VI; CF, caries free; dmfs, the decayed, missing and filled surfaces for deciduous teeth; dmft/DMFT, the decayed, missing, and filled teeth, related to deciduous/permanent tooth caries, respectively; ECC, early childhood caries; NR, not reported numerically; WSL, white spot lesion.

As shown in Figure 2, the results of the pooled data from seven studies with 411 participants (203 caries‐free) (Borghi et al., 2017; Frasseto et al., 2012; Kormi et al., 2020; Picco et al., 2017, 2019; de Sousa et al., 2021b) revealed that the activity of CA VI was significantly higher among participants with caries than among their caries‐free counterparts (SMD = 0.894, CI_95%_: 0.386 and 1.392, *Z* score = 3.519, *p* < .001; *τ* ^2^ = 0.361; *Q* value = 33.53; *df* = 6; *p* < .001; *I* ^2^ = 82%). As shown in Figure 3, the results of the pooled data from two studies with 141 participants (71 caries‐free) (Picco et al., 2017, 2019) revealed that the concentration of CA VI was significantly lower among participants with caries than among their caries‐free counterparts (SMD = −0.672, CI_95%_: −1.011 and −0.332, *Z* score = −3.878 *p* < .001; *τ* ^2^ = 0.00; *Q* value = 0.227; *df* = 1; *p* = .634; *I* ^2^ = 0%).

### 3.4 Publication bias

The funnel plot (Supporting Information: Figure 1) shows evidence of asymmetry in the included data, suggestive of a sort of publication bias. This bias was further quantitively confirmed using Egger's test, in which the two‐tailed *p* value was .04964.

### 3.5 Quality of the included studies

Out of the nine included studies, seven (Borghi et al., 2017; Frasseto et al., 2012; Kormi et al., 2020; Picco et al., 2017, 2019; de‐Sousa et al., 2021a; de Sousa et al., 2021b) had a low risk of bias, while the remaining two (Cardoso et al., 2017; Kivelä et al., 1999) had a moderate risk of bias (Table 3).

**Table 3:** Quality assessment of the studies by Newcastle–Ottawa Scale (NOS).

Study	Selection	Comparability	Exposure	Total score	Quality
Frasseto et al. (2012)	***	**	***	8	High
Cardoso et al. (2017)	**	‐	***	5	Moderate
Kormi et al. (2020)	***	**	***	8	High
Kivelä et al. (1999)	**	‐	***	5	Moderate
de‐Sousa et al. (2021a)	****	*	**	7	High
Picco et al. (2019)	****	*	**	7	High
Borghi et al. (2017)	****	*	**	7	High
Picco et al. (2017)	****	*	**	7	High
de Sousa et al. (2021b)	****	*	**	7	High

## 4 DISCUSSION

Dental caries involves a complex interaction over time among acid‐producing bacteria, fermentable carbohydrates, and many host factors, including dental and salivary characteristics. Risk factors for dental caries include physical, biological, environmental, behavioral, and lifestyle‐related variables such as high numbers of cariogenic bacteria, inadequate salivary flow, insufficient fluoride exposure, poor oral hygiene, inappropriate methods of feeding infants, high levels of sugar consumption, and low socioeconomic status. An integral function of salivary components is to maintain homeostasis in the oral environment, to provide a buffering capacity to neutralize acidic shifts in the oral environment and to protect the tooth surface from dental caries. CA VI is part of the defense system of saliva, increasing its buffering capacity by catalyzing the reaction of carbon dioxide. There is high individual variation in CA VI secretion into saliva in terms of its concentration and activity. It is paramount, therefore, to study the effect of such variation and their role as a predictor of dental caries. Various studies have investigated the effect of CA VI on dental caries incidence but reported conflicting results. Öztürk et al. studied young adults and found no association between dental caries and the CA VI concentration (Öztürk et al., 2008). However, other studies have reported an association between low CA VI concentrations and a higher caries index. A high concentration of CA VI is likely present in the oral environment when pH values are neutral, and higher activity of CA VI is expected when the oral environment becomes acidic. The current study reviewed the existing evidence of the role of CA VI concentrations and activity in dental caries.

Based on the current meta‐analysis, CA VI activity is higher, and CA VI concentrations are lower among patients with dental caries than among caries‐free subjects. Thus, the concentration and activity of CA VI may reflect completely different biological functions or, at a minimum, different stages of the same function. The salivary CA VI is likely being activated in individuals with active caries to slow and/or revert the caries process. The net result in a given individual with dental caries is high CA VI activity but lower CA VI concentration, and vice versa. Although such an inference is mainly based on two studies that measured both the concentration and activity of CA VI in the same subjects (Picco et al., 2017, 2019), the same can be deduced in the seven studies included in the meta‐analysis owing to the homogeneity of the main outcome (decreased CA VI activity) (Borghi et al., 2017; Frasseto et al., 2012; Kormi et al., 2020; Picco et al., 2017, 2019; de Sousa et al., 2021b). Taken together, the results of the current systematic review and meta‐analysis indicate that CA VI acts as a protective enzyme against the development of dental caries and suggest that CA VI might be utilized as a potential predictor of the existence and/or activity of dental caries.

The strengths of the current study include the following: First, this is the first comprehensive systematic review and meta‐analysis to assess the potential association between the activity and/or concentration of CA VI and dental caries. Second, our results were based on the qualitative and quantitative analysis of nine and seven studies (including recent studies), respectively. Third, the main outcomes were homogenous among the included studies. However, many limitations should be noted. First, few studies were included; additional, more robust, and methodologically sound primary studies are needed. Second, seven out of the nine studies included were conducted in Brazil, with many of the studies having overlapping authors, a matter that impacts the external validity (generalizability) of the results. Third, the measuring units were not standardized among studies, although this was accounted for in the meta‐analysis using the SMD. Fourth, and as indicated above, only two studies measured both the concentration and activity of CA VI in the same subjects (Picco et al., 2017, 2019). While this may weaken the validity of the inference described above, it strongly emphasizes the importance of measuring the concentration and activity of CA VI in future studies.

Given the results of this systematic review and meta‐analysis, and in light of the limitations indicated, we propose conducting studies to assess the clinical utility of CA VI as a predictor of the development and/or activity of dental caries and conducting studies on the clinical efficacy of adding CA VI into toothpaste formulas to prevent dental caries.

## 5 CONCLUSIONS

In conclusion, this meta‐analysis is one of the first to critically assess the role of the CA VI protein concentration and its activity in relation to dental caries. In light of the limitations of this systematic review, and according to our findings, we suggest that CA VI has lower concentrations and higher activity in patients with dental caries than in caries‐free individuals. Further studies are needed to determine the exact role of CA VI in dental caries.

## AUTHOR CONTRIBUTIONS

Rania Al‐Mahdi and Esam Halboub design the study. Rania Al‐Mahdi and Hesham Al‐Sharani searched and gathered the relevant articles. Hesham Al‐Sharani registered the project in the PROSPERO database. Rania Al‐Mahdi, Mohammed Al‐Haroni, and Esam Halboub drafted the manuscript. Rania Al‐Mahdi, Hesham Al‐Sharani, Mohammed Al‐Haroni, and Esam Halboub analyzed the data. The final manuscript is approved by all authors.

## CONFLICT OF INTEREST STATEMENT

The authors declare no conflict of interest.

## ETHICS STATEMENT

An ethics statement was not required for this study type, and no human or animal subjects or materials were used.

## Supporting information

Supporting information.

Supporting information.

## Data Availability

All data generated or analyzed during this study are included in this article. Further enquiries can be directed to the corresponding author.
