# Supplementary material for: Associations of the activity and concentration of carbonic anhydrase VI with susceptibility to dental caries: A systematic review and meta‐analysis
Source: Clin Exp Dent Res. 2023 Feb 23;9(2):358–67. doi: 10.1002/cre2.723 (PMC10098285; doi:10.1002/cre2.723)
Supplement: Supplementary file 1 — Supporting information. [file CRE2-9-358-s002.docx]

**Supplementary Table 1.** Excluded studies from the meta-analysis.

| **Author and year** | **Reason of exclusion** |
| --- | --- |
| Hou Wen *et al,* 2018 ^(Wen H, 2018)^ | The title addressed the relationship between carbonic anhydrase Ⅵ and caries, but the manuscript further explained the relationship between carbonic anhydrase Ⅵ and dental plaque. |
| Szabó *et al,* 1974 ^(Szabo, 1974)^ | The numerical data were not sufficient to be included in the meta-analysis. |
| Leinonen *et al,* 1999 ^(Leinonenet al., 1999)^ | The numerical data were not sufficient to be included in the meta-analysis. |
