# Supplementary figures and images for: Associations of the activity and concentration of carbonic anhydrase VI with susceptibility to dental caries: A systematic review and meta‐analysis
Source: Clin Exp Dent Res. 2023 Feb 23;9(2):358–67. doi: 10.1002/cre2.723 (PMC10098285; doi:10.1002/cre2.723)

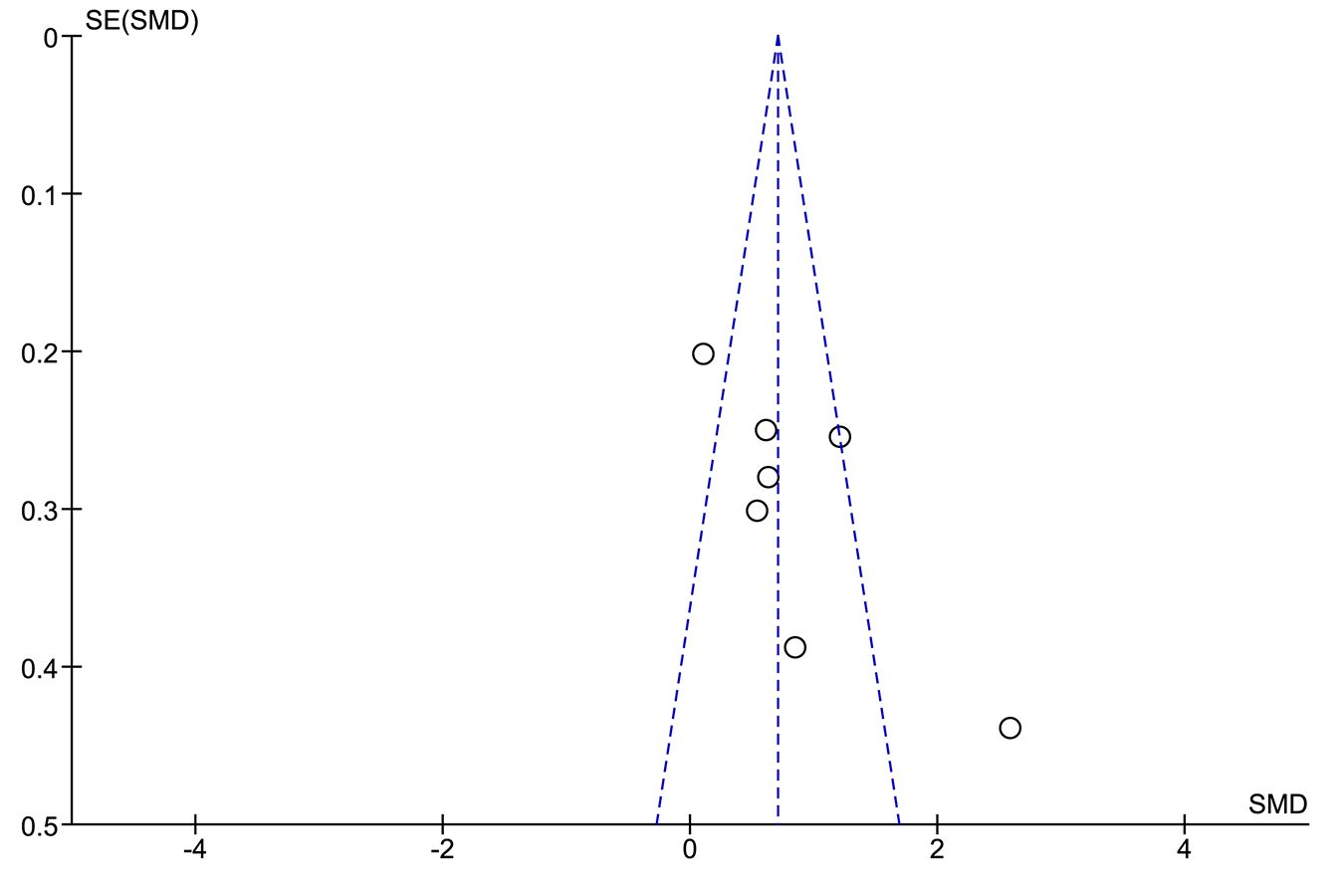


**Supplementary Figure 1**. The Funnel plot analysis of publications bias.

Supplement: Supplementary file 2 — Supporting information. [file CRE2-9-358-s001.docx]
